# The Relationship between Prebiotic Supplementation and Anthropometric and Biochemical Parameters in Patients with NAFLD—A Systematic Review and Meta-Analysis of Randomized Controlled Trials

**DOI:** 10.3390/nu12113460

**Published:** 2020-11-11

**Authors:** Ewa Stachowska, Piero Portincasa, Dominika Jamioł-Milc, Dominika Maciejewska-Markiewicz, Karolina Skonieczna-Żydecka

**Affiliations:** 1 Department of Human Nutrition and Metabolomics, Pomeranian Medical University in Szczecin, 71-460 Szczecin, Poland; ewa.stachowska@pum.edu.pl (E.S.); dmaciejewska.pum@gmail.com (D.M.-M.); karzyd@pum.edu.pl (K.S.-Ż.); 2 Clinica Medica “A. Murri”, Department of Biomedical Sciences & Human Oncology, University of Bari Medical School, 70124 Bari, Italy; piero.portincasa@uniba.it

**Keywords:** fiber, prebiotic, NAFLD, meta-analysis

## Abstract

We aim to systematically review the efficacy of prebiotics in reducing anthropometric and biochemical parameters in individuals with non-alcoholic fatty liver disease (NAFLD). A systematic search using PubMed/MEDLINE, Embase, clinicaltrials.gov, Cinahl, and Web of Science of articles published up to 20 March 2020 was performed for randomized controlled trials enrolling >20 adult patients. Random-effect meta-analysis for metabolic outcomes in NAFLD patients was performed for anthropometric data in addition to liver enzyme, carbohydrate, and lipid parameters. We found six trials (comprising a total of 242 patients) with NAFLD, with subjects aged 38–52 years. The mean time of fiber administration varied between 10 and 12 weeks. The main fiber types were psyllium (seeds or powder), *Ocimum basilicum* (seeds), and high-performance inulin and oligofructose powder at doses of either 10 or 16 g per day. The control group received either maltodextrin (powder or capsules) or crushed wheat (powder). Patients on the diet with added fiber had improvements in body mass index (BMI) (standardized mean difference (SMD) = −0.494, 95% confidence interval (CI): −0.864 to −0.125, *p* = 0.009); alanine aminotransferase (ALT) (SMD = −0.667, 95% CI: −1.046 to −0.288, *p* = 0.001); aspartate aminotransferase (AST) (SMD = −0.466, 95% CI: −0.840 to −0.091, *p* = 0.015); fasting insulin (SMD = −0.705, 95% CI: −1.115 to −0.295, *p* = 0.001); and homeostasis model assessment for insulin resistance (HOMA-IR) (SMD = −0.619, 95% CI: −1.026 to −0.211, *p* = 0.003). Hence, the results show that fiber supplements result in favorable changes as reflected in the measurement of anthropometric, metabolic, and liver-related biomarkers, i.e., body mass index (BMI), homeostasis model assessment for insulin resistance (HOMA-IR), insulin, alanine aminotransferase (ALT), and aspartate aminotransferase (AST). These effects suggest the potential benefits of fiber consumption for NAFLD populations. More prospective, controlled studies should be conducted to reveal specific details regarding the fiber type, dosage, and duration for optimal intervention.

## 1. Introduction

Chronic liver disease is most often caused by non-alcoholic fatty liver disease (NAFLD) [1]. The spectrum of NAFLD ranges from simply steatosis (i.e., non-alcoholic fatty liver, NAFL) when vesicular fat exceeds more than 5% of liver weight, to the progressive non-alcoholic steatohepatitis (NASH), with hepatocyte ballooning, inflammation, and fibrosis [1,2]. Globally, the estimated prevalence of NAFLD is about 25–35%, with the highest rates in Middle East and South American countries (approximately 30%). The majority of the NAFLD studies are based on data from North America, where NAFLD prevalence varies between 21% and 25%. In Europe, the prevalence of NAFLD is about 24% [3]. In the last two decades, NAFLD prevalence has increased in most populations worldwide, mirroring the rising trends in obesity. Meta-analyses demonstrated that the incidence in 2005 was 15%, whereas in 2010, it was equal to 25% [4].

Epidemiological risk factors for NAFLD include metabolic abnormalities such as obesity, diabetes, metabolic syndrome, poor socioeconomic conditions, and unfavorable lifestyles. Overall, the highest risk of NAFLD has been indicated for individuals with progressive metabolic disorders, including insulin resistance, dyslipidemia, and visceral fat accumulation [5]. Additionally, genetic predisposition involving adiponutrin, for example, is another risk factor [2].

As a result of the major impact of improper lifestyles on the pathogenesis of NAFLD, either modifications of dietary habits associated with weight loss or ideal weight maintenance and regular physical activity are advisable [6,7,8].

A reduction in body mass by at least 5% contributes to an improvement of histological hepatitis (without influence on fibrosis severity), whereas a decrease in body mass by more than 7% of initial weight significantly alleviates NASH [9]. A 30% cutoff of total daily caloric intake is recommended for body mass reduction (with respect to total daily energy expenditure (TDEE)) [10], which means a decrease in calorie intake by 750–1000 kcal/day (in relation to TDEE in real life) [11,12]. Therefore, the ideal diet should be hypocaloric and Mediterranean (MD) [10]. The choice of MD is not accidental, since MD is not only a nutrition pattern and, in fact, it denotes attitude toward life and ability to make the right food choices (based on healthy and local products such as vegetables, fruits, unrefined grains, legumes, fermented milk drinks, aquaculture products, etc.) [5,13,14]. In general, the concept of MD includes a high consumption of fresh, low-processed plant products rich in antioxidants and good-quality plant fiber, which makes it a diet rich in fiber [15].

Fiber is an important part of a healthy diet [16]. Fiber reduces hunger and modulates satiety as well as provides proper gastrointestinal motility, preventing constipation [17,18,19]. In addition, fibers are substrates used by gut microbiota for producing short fatty acids, namely acetate, propionate, and butyrate, which contribute to decreased luminal pH, increased motility, and enterocyte function. Fiber acts as a prebiotic, with beneficial effects on host health, i.e., by regulating the gut–brain axis (e.g., by suppressing appetite) [20] and, in this way, it also regulates body mass [21,22,23].

Results of reports published so far indicate the undeniable health benefits associated with an increased intake of dietary fiber, including reduced risk of obesity, diabetes, coronary heart disease, and metabolic disorders [24]. Dietary fiber intake differs distinctly across industrialized and unindustrialized parts of the world [25]: on average, adults consume 12–18 g/day of dietary fiber in the United States [26] and 16–29 g/day in Europe [27]. According to the EFSA (European Food Safety Authority), adequate fiber intake corresponds to at least 25 g per day [27]. Various types of plants such as psyllium, barley bran, and oat bran contain mostly soluble fiber and have been demonstrated to improve blood lipid levels, whereas insoluble fibers, e.g., bran, are typically linked to laxative properties and reduced all-cause mortality [28,29].

Low-fiber intake in Western countries is associated with changes in the gut microbiota, and abnormal microbiota composition might pave the way and contribute to chronic metabolic diseases, such as obesity, type 2 diabetes, cardiovascular disease, and colon cancer [25]. Data concerning the protective role of fiber consumption in NAFLD have so far been lacking. To date, one meta-analysis [30] and three systematic reviews (published between 2012–2015) [31,32,33] evaluating the efficacy of both prebiotics and probiotics have been published. Hence, here, we conduct the first systematic review and meta-analysis of studies with fiber added to diet in NAFLD subjects. We tested the hypothesis that fiber supplementation for a certain time would be superior to placebo and result in greater improvements in terms of body mass and biochemical parameters of NAFLD patients.

The effect of dietary fiber supplementation was evaluated based on several distinct parameters of liver function (i.e., alanine aminotransferase (ALT), aspartate aminotransferase (AST), γ-glutamyltransferase (GGT)) and of metabolic profiles (i.e., homeostasis model assessment for insulin resistance (HOMA-IR) index, blood insulin level, blood glucose level, blood lipid profile (triglycerides (TAG), cholesterol (CHOL), low density lipoprotein cholesterol (LDL-chol), high density lipoprotein cholesterol (HDL-chol)), and through anthropometric data (weight, waist–hip ratio (WHR), percentage body fat (PBF), trunk mass body fat (MBF), lean body mass (LBM), soft lean mass (SLM)).

## 2. Materials and Methods

### 2.1. Search Strategy and Inclusion Criteria

We used the preferred reporting items for systematic reviews and meta-analyses (PRISMA) guidelines [34] to perform this systematic review and meta-analysis of randomized controlled trials (RCTs). The literature search was conducted by two independent authors (ES, DMM) using 5 databases (PubMed/MEDLINE, Embase, clinicaltrials.gov, Cinahl, Web of Science) to identify trials published up to 20 March 2020. The following search strings were established in the search process:

PubMed/Cinahl/Web of Science: (prebiotic OR fiber) AND (NAFLD OR “non-alcoholic fatty liver” OR fibrosis OR cirrhosis OR steatohepatosis) AND (biopsy OR “hemoglobin A1C” OR HbA1C OR glucose OR hyperglycemia OR weight OR obesity OR obese OR overweight OR over-weight OR weight-gain OR metabolic OR metabolism OR cardiometabolic OR cholesterol OR triglycerides OR dyslipidemia OR lipid OR steatosis OR ALT OR AST OR GGTP OR HOMA OR “HOMA-IR” OR “hyaluronic acid”) AND (RCT OR random* OR placebo*).”

Embase: (“nonalcoholic fatty liver”/exp OR “nafld (nonalcoholic fatty liver disease)” OR “non alcoholic fatty liver disease” OR “non alcoholic hepatosteatosis” OR “non alcoholic liver steatosis” OR “non-alcoholic fld” OR “non-alcoholic fatty liver” OR “non-alcoholic fatty liver disease” OR “non-alcoholic hepatic steatosis” OR “nonalcoholic fld” OR “nonalcoholic fatty liver” OR “nonalcoholic fatty liver disease” OR “nonalcoholic hepatic steatosis” OR “nonalcoholic hepatosteatosis” OR “nonalcoholic liver steatosis” OR “liver fibrosis”/exp OR “fibrosis, liver” OR “fibrous hepatic disease” OR “hepatic fibrosis” OR “liver fibrosis” OR “liver periportal fibrosis” OR “periportal fibrosis” OR “liver cirrhosis”/exp OR “cirrhosis” OR “cirrhosis hepatis” OR “cirrhosis, liver” OR “cryptogenic liver cirrhosis” OR “dietary cirrhosis” OR “dietary liver cirrhosis” OR “hepatic cirrhosis” OR “liver cirrhosis” OR “postnecrotic liver cirrhosis”) AND (“prebiotic agent”/exp OR “prebiotic” OR “prebiotic agent” OR “prebiotics” OR “fiber”/exp OR “fiber” OR “fibre”) AND “placebo”/exp AND (“glycosylated hemoglobin”/exp OR “glycated haemoglobin” OR “glycated hemoglobin” OR “glycated hemoglobin a” OR “glycohaemoglobin” OR “glycohemoglobin” OR “glycosyl haemoglobin” OR “glycosyl hemoglobin” OR “glycosylated haemoglobin” OR “glycosylated hemoglobin” OR “glycosylhaemoglobin” OR “glycosylhemoglobin” OR “glycosylised haemoglobin” OR “glycosylized hemoglobin” OR “haemoglobin a1” OR “haemoglobin a 1” OR “haemoglobin a, glycosylated” OR “haemoglobin ai” OR “haemoglobin alpha 1” OR “haemoglobin glycoside” OR “haemoglobin glycosylation” OR “hemoglobin a, glycosylated” OR “hemoglobin glycoside” OR “glucose”/exp OR “glucose” OR “obesity”/exp OR “adipose tissue hyperplasia” OR “adipositas” OR “adiposity” OR “alimentary obesity” OR “body weight, excess” OR “corpulency” OR “fat overload syndrome” OR “nutritional obesity” OR “obesitas” OR “obesity” OR “overweight” OR “body weight”/exp OR “body weight” OR “total body weight” OR “weight, body” OR “body weight gain”/exp OR “body weight gain” OR “body weight increase” OR “weight gain” OR “weight increase” OR “cardiometabolic disease”/exp OR “cholesterol”/exp OR “3 hydroxy 5 cholestene” OR “3beta hydroxy 5 cholestene” OR “3beta hydroxycholest 5 ene” OR “5 cholesten 3beta ol” OR “beta cholesterol” OR “cholest 5 en 3beta ol” OR “cholest 5 ene 3 ol” OR “cholesterin” OR “cholesterine” OR “cholesterol” OR “cholesterol release” OR “dythol” OR “nsc 8798” OR “triacylglycerol”/exp OR “acylglycerol, tri” OR “fatty acid triglyceride” OR “triacyl glyceride” OR “triacylglycerol” OR “triglyceride” OR “triglycerides” OR “tryglyceride” OR “lipid”/exp OR “lipid” OR “lipid extract” OR “lipids” OR “lipids and antilipaemic agents” OR “lipids and antilipemic agents” OR “aminotransferase”/exp OR “amino transferase” OR “aminotransferase” OR “aminotransferases” OR “transaminase” OR “transaminases” OR “homa ir” OR “hyaluronic acid”/exp).

ClinicalTrials.gov: Prebiotic AND NAFLD

Only English language and human studies were included in the review.

The following inclusion criteria were applied:

(i) randomized controlled trial
(ii) patients with confirmed NAFLD
(iii) studies enrolling >20 patients
(iv) treatment with prebiotic (soluble fiber or insoluble fiber) in the form of a supplement, e.g., pills, powder, etc.
(v) randomization to prebiotic vs. placebo/other prebiotic/probiotic/synbiotic/no intervention (OPEN LABEL)
(vi) studies reporting at least one of the following outcomes: available meta-analyzable change score/endpoint data on steatosis, alanine aminotransferase, aspartate aminotransferase, γ-glutamyltransferase, HOMA-IR, blood insulin level, blood glucose level, lipids profile (triglycerides, cholesterol, LDL-chol, HDL-chol), anthropometric data (BMI, weight, waist–hip ratio (WHR), percentage body fat (PBF), trunk mass bod fat (MBF), lean body mass (LBM), soft lean mass (SLM)).

Studies involving patients suffering from hepatitis B virus (HBV) or hepatitis C virus (HCV) infection, ≤18 y or using co-interventions (i.e., herbal and pharmaceutical preparations) were excluded. Data from more than two-arm studies were evaluated separately for comparators [35].

### 2.2. Data Extraction

The database search process was done in accordance with the PRISMA diagram [36]. This step was performed by two independent reviewers (ES, DMM), and inconsistencies were resolved by mediators (DJM, KSZ). The collected data were as follows: publication year, study location, sponsorship, blinding, setting, focus of the study, patient, intervention, and comparator characteristics. Characteristics of the analyzed studies are presented in Table 1.

### 2.3. Outcomes

Co-primary outcomes were liver enzymes alanine aminotransferase (ALT), aspartate aminotransferase (AST), γ-glutamyltransferase, HOMA-IR index, blood insulin level, blood glucose level, blood lipid profile (triglycerides, cholesterol, LDL cholesterol, HDL cholesterol) and anthropometric data (BMI, weight, waist–hip ratio (WHR), percent body fat (PBF), trunk mass bod fat (MBF), lean body mass (LBM), soft lean mass (SLM)).

### 2.4. Data Synthesis and Statistical Analysis

A random-effects meta-analysis [42] of outcomes for which ≥2 studies contributed data was conducted using Comprehensive Meta-Analysis V3 (http://www.meta-analysis.com) software. We explored study heterogeneity using the chi-square test of homogeneity. In the case of *p* < 0.05, we qualified studies as significantly heterogenous. All analyses were two-tailed with alpha = 0.05.

We calculated pooled standardized mean difference (SMD) or, where applicable, difference in means (DM) in endpoint scores to assess group differences in continuous outcomes. Categorical outcomes were also analyzed by calculating the pooled risk ratio (RR) using endpoint scores. Finally, we inspected funnel plots and used Egger’s regression test [43] to quantify whether publication bias could have influenced the results.

### 2.5. Risk of Bias

The bias for each included study was assessed by two independent investigators (ES and DJM) in accordance with guidelines of the Cochrane Collaboration’s tool [44]. Within each bias category (selection, performance, detection, attrition, and reporting) of the assessment tool, the level of bias was rated as “low risk” or “high risk” or “unclear risk”. The risk of bias (RoB) was evaluated based on the assumption that the higher number of low-risk-of-bias assessments, the greater the quality of the study.

## 3. Results

### 3.1. Search Results

**Figure 1:**
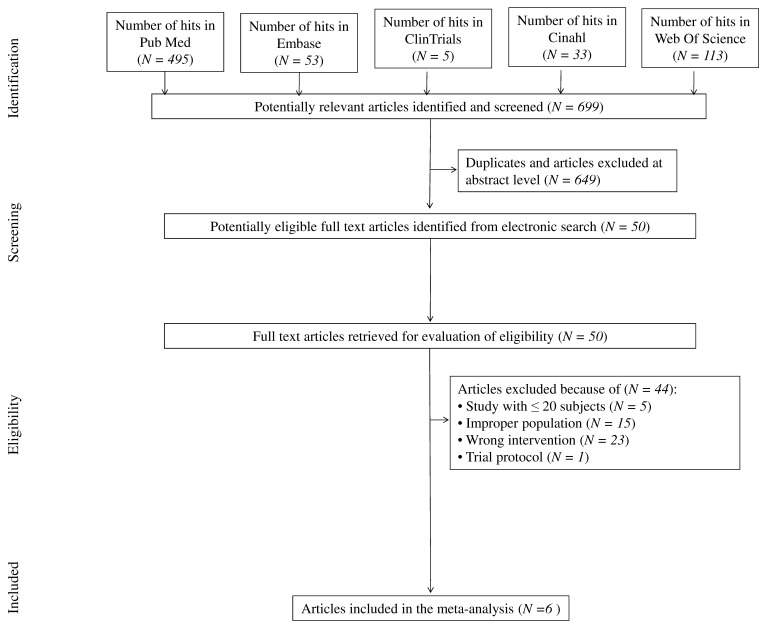
Study flow chart.

The search strategy identified a total of 699 published articles. The vast majority of studies (N = 649) were excluded for various reasons (i.e., being duplicates and/or after evaluation on the title/abstract level). After reviewing the full text of 50 studies, only six full-text articles met the inclusion criteria and were eligible for meta-analysis. The reasons for exclusion were overly small sample size (<20 patients) (N = 5), patients <18 years old, or with diagnosed hepatitis B/C virus or with diagnosed hepatic cirrhosis or without NAFLD diagnosis confirmation (N = 15), and wrong intervention (N = 23). We also excluded one study protocol. In three articles included in the present meta-analyses, the same study cohort was reported with identical numbers of patients in either the intervention or control groups also in addition to some results. However, in the present meta-analysis, these data were not triplicated. The study flow chart is depicted in Figure 1.

### 3.2. Study, Patient and Treatment Characteristics

Six full-text articles (N = 6) were included [35,37,38,39,40,41], although three described the same study population [39,40,41]; thus the numbers of studies and interventions were four and six, respectively. Two trials were single-blind [35,37], and the other two were double-blind [38,39,40,41]. All studies were conducted in Iran and involved both men and women, but gender-specific results were missing. The following different types of prebiotics were administered: psyllium (seeds or powder) [35,37], *Ocimum basilicum* (seeds) [35], oligofructose powder [38] and high-performance inulin (inulin HP) powder [39,40,41] at doses of either 10 g [35,37,39,40,41] or 16 g [38] per day. In the control group, maltodextrin (powder or capsules) [38,39,40,41], crushed wheat (powder) [37], or no comparator were given [35]. Additionally, in two studies, patients received diet and physical activity recommendations [37,38], while in the other two, no additional intervention was mentioned [35,39,40,41]. The duration of treatment ranged from 10 [37] to 12 weeks [35,38,39,40,41]. A total number of 242 patients with diagnosed (with ultrasonography and/or AST and/or ALT level) NAFLD, aged 38–52 years, participated in these six interventions, and the mean number of randomized and analyzed patients per trial was 76 and 60, respectively. The main characteristics of the included studies are presented in Table 1.

### 3.3. Risk of Bias

All studies included in the meta-analysis were of high quality (>3 low-risk assessments) with the highest number being 5 [35,37,38,40,41] and the lowest being 4 [39] out of a possible 7. Two studies were single-blinded [35,37]. Both random sequence generation and allocation concealment were sufficiently described and assessed as low risk in all studies. The highest number of high-risk assessments was recorded in reporting bias [35,38,40,41] and other bias [35,37,38,39,40,41]. The overall risk of bias, depicted as the number of low-risk-of-bias assessments of each study, is presented in Table 1.

### 3.4. Fiber Effects on NAFLD-Related Parameters

**Figure 2:**
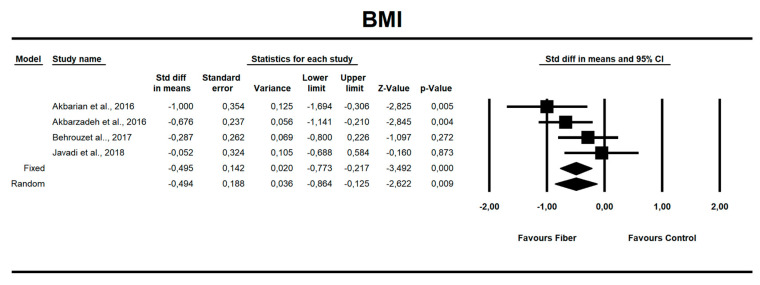
Effect size and standardized mean difference (SMD) for body mass index (BMI) in people supplementing fiber vs. controls. Q = 5.108, df(Q) = 3, *p* = 0.164, I-squared = 41.273.

**Figure 3:**
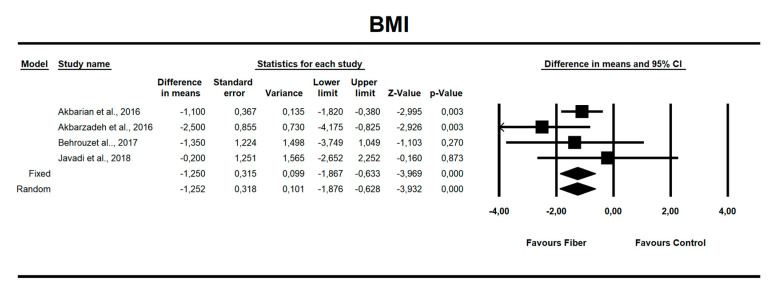
Effect size and difference in means (DM) for BMI in people supplementing fiber vs. controls. Q = 3.018, df(Q) = 3, *p* = 0.389, I-squared = 0.588.

**Figure 4:**
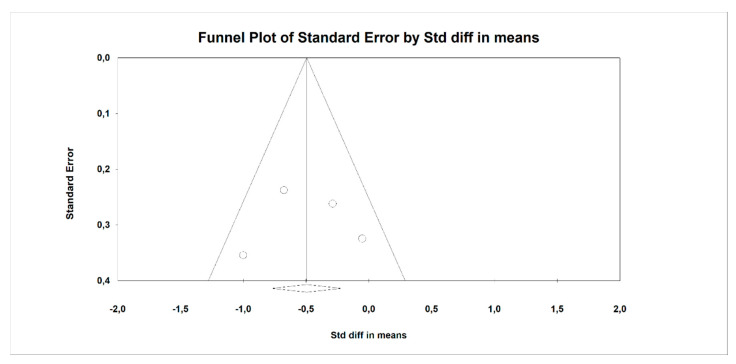
Funnel plot for endpoint BMI (standardized mean difference) in the present meta-analysis. Egger’s test: *p* = 0.957.

**Figure 5:**
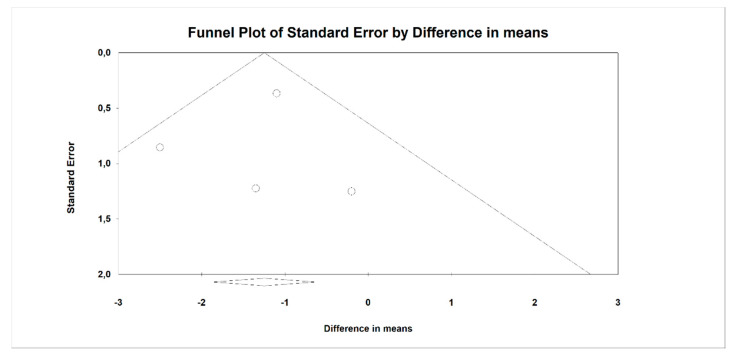
Funnel plot for endpoint BMI (difference in means) in the present meta-analysis. Egger’s test: *p* = 0.827.

Using random-effects weights, we found that BMI, ALT, AST, HOMA-IR, and insulin were significantly affected by fiber intake. The standardized mean difference for BMI (with more than two studies contributing data) was −0.494 with a 95% confidence interval of −0.864 to −0.125 (z = −2.622, *p* = 0.009; Figure 2). In case of difference in means, it was equal to −1.252 with a 95% confidence interval of −1.876 to −0.628 (z = −3.932; *p* = 0.000, Figure 3). In both cases, Egger’s tests provided no evidence of bias in the estimations (SMD: *p* = 0.957; DM: *p* = 0.827; Figure 4 and Figure 5).

**Figure 6:**
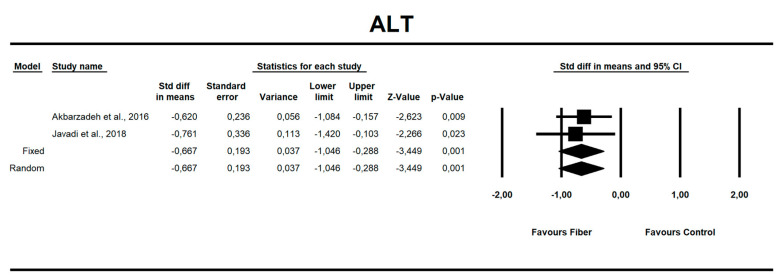
Effect size and standardized mean difference (SMD) for alanine aminotransferase (ALT) in people supplementing fiber vs. controls. Q = 0.118, df(Q) = 1, *p* = 0.732, I-squared = 0.0.

**Figure 7:**
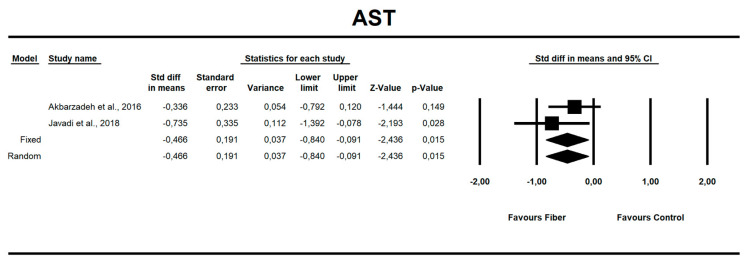
Effect size and standardized mean difference (SMD) for aspartate aminotransferase (AST) in people supplementing fiber vs. controls. Q = 0.958, df(Q) = 1, *p* = 0.328, I-squared = 0.0.

**Figure 8:**
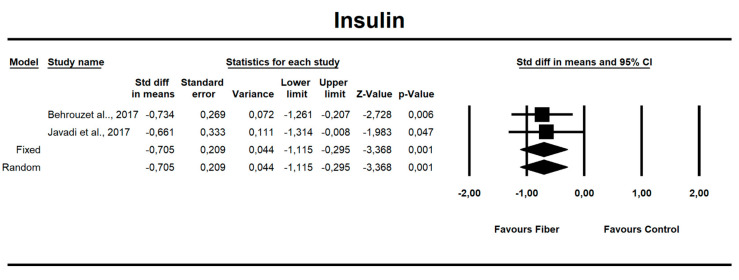
Effect size and standardized mean difference (SMD) for insulin in people supplementing fiber vs. controls. df(Q) = 1, *p* = 0.864, I-squared = 0.0.

**Figure 9:**
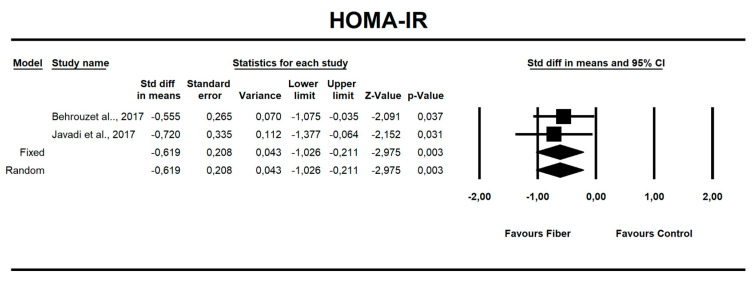
Effect size and standardized mean difference (SMD) for homeostasis model assessment for insulin resistance (HOMA-IR) in people supplementing fiber vs. controls. Q = 0.150, df(Q) = 1, *p* = 0.698, I-squared = 0.0.

Using only SMD effect size, we found that along with the fiber intake ALT (−0.667; 95% CI of −1.046 to −0.288; z = −3.449, p = 0.001; Figure 6), AST (−0.466; 95% CI of −0.840 to −0.091; z = −2.436, *p* = 0.015; Figure 7), insulin (−0.705; 95% CI of −1.115 to −0.295; z = −3.368, *p* = 0.001; Figure 8), and HOMA-IR (−0.619; 95% CI of −1.026 to −0.211; z = −2.975, p=0.003; Figure 9) values decreased. For these results, only two studies provided data; thus, this was not enough to conduct publication bias analyses.

Other outcomes did not yield statistically significant results. In addition, in the case of post-intervention energy intake, Egger’s test suggested publication bias (*p* = 0.035) (Figures S1–S15).

Regarding heterogeneity, in a few calculations, i.e., post-intervention carbohydrate intake, LBM, MBF, PBF, SLM, and body weight, all of which were not significantly affected by fiber ingestion, significant heterogeneity was demonstrated (see Supplementary Figures S1, S4–S6, S8 and S9).

## 4. Discussion

### 4.1. Body Mass Index

Achieving and maintaining the ideal body weight (normal BMI) is one of the main therapeutic strategies in patients with metabolic disorders, including NAFLD [9]. This apparently simple goal can be difficult to reach and maintain, including for NAFLD patients. Maintaining healthy lifestyles and diet are unachievable for most patients. Only 50% of patients tend to maintain weight reduction at 7% after 12 months of therapy [9]. Therefore, a practical issue is to search for ingredients that can be easily added to the regular diet that can help to effectively sustain body weight reduction without any side effects.

The ultimate role of fiber as a dietary component in NAFLD patients has not been properly addressed so far, although fiber has been described for years as having a tremendous effect on weight reduction [21]. In most recent publications, fiber was studied together with probiotics, which does not allow for a clear picture of its sole influence on selected NAFLD-linked parameters [30,31,32]. The very few studies focused on fiber (without probiotics) suggest a weak role of fiber in weight reduction among NAFLD individuals [35,37,38,39,40,41]. The fiber acts as a prebiotic and supports the growth of commensal microbiota. Loman et al. [30] described such therapy as of a microbial type and highlighted that BMI significantly decreased after the intervention (by −0.37 kg/m^2^; *p* < 0.001). Other meta-analyses focused on body mass parameters and fiber, and all demonstrated that subjects consuming fiber or high-fiber diets achieved (in most studies) a significant reduction in body weight [45,46,47]. In their meta-analysis, Thomson and colleagues postulated that an isolated supplementation of soluble fiber is linked to an improvement in anthropometric and metabolic outcomes in overweight and obese adults [48].

What is the likely mechanism of weight reduction after increasing the amount of fiber in the diet? First of all, fiber increases the feeling of satiety after a meal due to gastric distention and the activation of afferent vagal signals; second, as an indigestible component of food, fiber regulates the defecation rhythm; third, it can selectively influence the growth of intestinal bacteria [49]. Effects on microorganisms may be crucial since gut microbiota provide various benefits for host health, including (among other things) the harvesting energy from the diet or maintenance of mucosal barrier integrity [49,50]. It seems that modulation of the composition and function of intestinal bacteria can play a role in nutrition and health [23].

The first evidence for a putative role of gut microflora in NAFLD was suggested more than 20 years ago, and a growing number of studies are confirming this phenomenon [32,33,50,51,52]. A meta-analysis of 21 randomized controlled trials found that fiber added to the diet restores bacterial homeostasis and thus promotes weight reduction [45]. Intestinal bacteria (*Lactobacillus* and *Bifidobacterium* genera) have a positive effect on the reduction of the weight through a few mechanisms [45,53,54]: (i) reduction of inflammation and thus improvement of hypothalamic sensitivity to insulin; (ii) biotransformation of primary to secondary bile acids, which play an important role as signaling agents on both nuclear and membrane-associated intestinal and extra-intestinal receptors in enterohepatic circulation; (iii) stimulation of the secretion of certain intestinal hormones—peptide YY (PYYY) and glucagon-like peptide-1 (GLP-1) and GLP-2 (GLP-1 also suppresses the appetite by delaying gastric emptying and centrally promoting satiation) [54,55]; (iv) downregulating the expression of fasting-induced adipocyte factor (Fiaf) from gut epithelial cells, thus resulting in the degradation of lipoproteins and the deposition of free fatty acids in adipose tissue [56].

Results included in our meta-analysis are supported by high-quality clinical studies, and the type of fiber (used in most of these studies) has been widely exploited. Plantago seeds used by Akbarian and colleagues [35] (reviewing studies of other researchers) managed to decrease total fat intake in diet, which may be a useful supplement in weight control diets [57], and improve bowel movement [58] as well plasma lipid status in men with ischemic heart disease [59]. Plantago powder used by Akbarzadeh [37] in another study improved BMI [60], reduced fasting (but not postprandial) plasma insulin [61], and reduced total serum cholesterol levels, low-density lipoprotein cholesterol levels, and the ratio of LDL cholesterol to high-density lipoprotein cholesterol [62]. Oligofructose (Orafti^®^ P95) powder applied by Behrouz [38] provided a laxation effect without causing gastrointestinal distress for healthy participants with irregularity associated with low dietary fiber intake [63]. Oligofructose used separately as a prebiotic (administered as high-oligofructose granola bar) lowered appetite [64], reduced the postprandial blood glucose response to foods (yogurt drink containing oligofructose) [65], and at a dose of 16 g/d, acted as an effective reductor for energy intake (possibly through increasing GLP-1 and PYY secretion) [66].

Inulin used by Javadi et al. [39,40,41] is the best of the tested prebiotic in terms of health benefits. Meta-analyses by Rao and colleagues [67] have shown that inulin-type carbohydrates can ameliorate insulin resistance in type 2 diabetes mellitus and obese individuals. According to Liu and colleagues [68], inulin-type fructans may improve the lipid profile (LDL-c reduction in the general population and HDL-c in type 2 diabetes mellitus (T2DM) patients) and glucose control (in T2DM subgroup). Brighenti also showed that dietary inulin-type fructans significantly reduced serum triacylglycerols (by influencing colonic fermentation and/or incretin release from the distal gut) [69]. Inulin has a significant overall effect on stool frequency (DEM = 0.69, 95% CI: 0.04, 1.34), consistency, and transit time, thereby reducing the constipation that accompanies obesity [70].

### 4.2. Glycemia

There is strong epidemiologic evidence that dietary fiber intake is protective against hyperglycemia and insulinemia [48,71]. Our results show that fiber has a small but positive effect on fasting insulin levels (SMD = −0.705, 95% CI: −1.15 to −0.29, *p* = 0.001), and on HOMA-IR (SMD = −0.619, 95% CI: −1.026 to −0.211, *p* = 0.003) but not for fasting or postprandial glycemia.

The results obtained from this meta-analysis support the concept that fiber has a positive effect on glucose metabolism, although our meta-analyses could only focus on poor-quality clinical trials to support these results in NAFLD patients. Meanwhile, Thompson and colleagues [48] have shown in their meta-analysis comprising overweight and obese individuals that soluble fiber reduces fasting glucose by 0.17 mmol/L and insulin by 15.88 pmol/L as compared to placebo. Jovanovski et al. [71] also demonstrated in a type 2 diabetes cohort that viscous fiber supplements at a median dose of ≈13.1 g/day improved glycemia by reducing fasting blood glucose and HOMA-IR. Li-Xia He and colleagues indicated that fiber from whole oats and oat bran is associated with lower fasting glucose and fasting insulin in T2D, hyperlipidemic, and overweight subjects [72].

### 4.3. Hepatic Enzymes

In our meta-analysis, the use of fiber favored improvement through an increased enzyme secretion of ALT (MD)= −6.67 (95% CI; *p* = 0.001) and AST (MD) = −0.466 (95% CI; *p* = 0.015). In a meta-analysis by Loman et al. [30], who analyzed both pre- and probiotics in NAFLD patients, results were similar: microbial-based therapies favored an increased secretion of hepatic enzymes (ALT (MD) = −6.9 U/L, AST (MD) = −4.6 U/L, γ-GTP (MD) = −7.9 U/L, *p* < 0.001). Behrouz et al. [73], in a double-blind randomized clinical trial, noted that prebiotic (oligofructose) supplementation causes a significant decrease in ALT and AST levels compared to the control group (placebo). This observation can be explained in terms of weight loss: weight reduction by 5–10% results in a 20–80% decrease in serum aminotransferase activity [10,74].

## 5. Limitations

The limitations of this meta-analysis include (i) a relatively small number of high-quality double-blinded studies comparing prebiotic intervention to controls with a wide range within the number of participants preceded by no sample size calculations; (ii) heterogeneous study inclusion criteria (various ages, profession of participants, and dietary and physical activity add-on interventions); (iii) the association between the prebiotic effect in relation to supplement dose and treatment duration was not analyzed as too few studies were included; (iv) the studies lacking a proper calculation of the amount of fiber in a regular diet; (v) the diagnostic methods used in included studies to identify NAFLD (ultrasonography and/or AST and/or ALT level). This latter aspect is a great weakness in most studies dealing with prebiotic supplementation.

## 6. Conclusions

Although a relevant number of studies dealing with fiber supplementation and NAFLD within a metabolic background have appeared in the literature, stringent criteria have resulted in a decreased number of available associated trials. The meta-analysis of the few available studies indicate that fiber supplements might provide benefits to NAFLD populations based on measurements of at least some of the metabolic and liver-related biomarkers (i.e., BMI, ALT, and AST outcomes).

Our meta-analysis indicates the need for randomized controlled trials based on strict inclusion criteria and homogenous intervention protocols both in healthy adults and those with NAFLD, which would allow for clearly defining the impact of prebiotics on anthropometric and biochemical parameters and to develop guidelines regarding their intake.

## Figures and Tables

**Table 1:** Study characteristics.

No	Study Characteristics (First Author, Year, Country)	Study Design			Intervention					Patient Characteristics			Dietary Habits	
		Blinding/ ROB	Focus of Study	NAFLD Diagnosis	Prebiotic		Comparator		Additional Intervention	R;A (*n*)	Age (Years) (Mean ± SD)	Male (%)	Energy (kcal/day) at Baseline ** (Mean ± SD) T;C	Energy (kcal/day) after Intervention ** (Mean ± SD) T;C
					Specification	Oral Dose/Duration	Type	Oral Dose						
1a	Akbarian et al., 2016, Iran [35]	SB/H	anthropometric measures in nonalcoholic fatty liver patients	ultrasonography	*Ocimum basilicum* (OB); seeds	10 g/day/12 weeks	none	N/A	none	55;36	52.2 ± 3.9	25 ^†^	1844.3 ± 116.9; 2010.8 ± 131.4	nd
1b	Akbarian et al., 2016, Iran [35]				*Plantago psyllium* (PP); seeds					54;35	48.4 ± 2.9	22.9 ^†^	1794.2 ± 116; 2010.8 ± 131.4	
1c	Akbarian et al., 2016, Iran [35]				*Plantago psyllium* (PP) and *Ocimum basilicum* (OB); seeds					53;37	51.3 ± 3.0	18.9 ^†^	2215.1 ± 85.6; 2010.8 ± 131.4	
2	Akbarzadeh et al., 2016, Iran [37]	SB/H	anthropometric measurements, body composition and liver enzymes in overweight or obese adults with NAFLD	Physical examination and/or ALT > 40 IU/L and/or elastometry value > 4 kPa in FibroScan (FibroScan 402, Paris, France)	Psyllium; powder	10 g (2 × 5 g)/day/10 weeks	placebo—crushed wheat (powder)	10 g (2 × 5 g)/day	physical activity and weight loss diet recommendation *	80;75	45 ± 14.7	46.7 ^†^	2044.8 ± 527.8; 2449.7 ± 778.4	1601.3 ± 624.8; 1732.9 ± 468.3
3	Behrouz et al., 2017, Iran [38]	DB/H	adiokines and glycemic parameters in the patients with NAFLD	ultrasonography and ALT > 1.5 × upper limit of normal	ORAFTI P95-oligofructose powder, (BENEO, Belgium) ^$^; capsules	16 g (2 × 8 g)/day/12 weeks	placebo—maltodextrin (capsules)	16 g (2 × 8g)/day	physical activity and weight loss diet recommendation ^#^	70;59	38.4 ± 9.7	69.5 ^†^	2527.9 ± 681.7; 2417.1 ± 706.5	1917.2 ± 384.6; 1909.9 ± 422.1
4	Javadi et al., 2018, Iran [39]	DB/H	oxidative stress and inflammatory markers in patients with NAFLD	ultrasonography and ALT > 37 units/L and AST > 40 units/L	Inulin HP (Sensus, Borchwerf, 34704 RG Roosendaal, The Netherlands) ^$^; powder (sachet)	10 g (2 × 5 g)/day/12 weeks	placebo—maltodextrin (powder, sachet)	10 g (2 × 5 g)/day	none	42/38	40.4 ± 9.7	76.3	2296 ± 282; 2158 ± 464	2244 ± 174; 2080 ± 408
5	Javadi et al., 2017, Iran [40]	DB/H	liver function tests in patients with NAFLD	ultrasonography and liver enzymes tests (cutoff values: AST 31 IU/L, ALT 30 IU/L)										nd
6	Javadi et al., 2017, Iran [41]	DB/H	lipid profile and insulin resistance factors in NAFLD patients	ultrasonography and ALT > 37 units/L and AST > 40 units/L										

^†^ % of analyzed patients; * for treatment and control group: regular exercise for at least 30 min/3 times per week and a weight loss diet (calorie restriction less than 30% total calorie need, total dietary fat <30%, saturated fats <10%, carbohydrate 40%–54% of total calorie need; ** 72 h food dietary recall; ^#^ according to the Practical Guide Identification, Evaluation, and Treatment of Overweight and Obesity in Adults from National Institute of Health; ^$^ manufacturer data; A—number of analyzed patients; ALT—alanine aminotransferase; AST—aspartate aminotransferase; C—control group; DB—double-blinded; H—high-quality study; inulin HP—high performance inulin; IU/L—international unil/liter; N/A—not applicable; NAFLD—non-alcoholic fatty liver disease; nd—no data; ORAFTI—trademark; R—number of randomized patients; ROB—risk of bias; SB—single-blinded; SD—standard deviation; T—treatment group.

## Supplementary Materials

The following are available online at https://www.mdpi.com/2072-6643/12/11/3460/s1. Figure S1. Effect size and standardized mean difference for carbohydrate intake in people supplementing fiber vs. controls. Q = 10.836, df(Q) = 1, *p* = 0.001, I-squared = 90.771; Figure S2. Effect size and standardized mean difference for energy intake in people supplementing fiber vs. controls. Q = 3.560, df(Q) = 2, *p* = 0.169, I-squared = 43.825; Figure S3. Effect size and standardized mean difference for fat intake in people supplementing fiber vs. controls. Q = 1.404, df(Q) = 1, *p* = 0.236, I-squared = 28.724; Figure S4. Effect size and standardized mean difference for LBM in people supplementing fiber vs. controls. Q = 45.474, df(Q) = 2, *p* = 0.00, I-squared = 95.602; Figure S5. Effect size and standardized mean difference for MBF in people supplementing fiber vs. controls. Q = 9.158, df(Q) = 2, *p* = 0.01, I-squared = 78.161; Figure S6. Effect size and standardized mean difference for PBF in people supplementing fiber vs. controls. Q = 20.996, df(Q) = 2, *p* = 0.00, I-squared = 90.474; Figure S7. Effect size and standardized mean difference for protein intake in people supplementing fiber vs. controls. Q = 1.190, df(Q) = 1, *p* = 0.275, I-squared = 15.955; Figure S8. Effect size and standardized mean difference for SLM in people supplementing fiber vs. controls. Q = 46.227, df(Q) = 1, *p* = 0.00, I-squared = 97.837; Figure S9. Effect size and standardized mean difference for body weight in people supplementing fiber vs. controls. Q = 25.572, df(Q) = 3, *p* = 0.00, I-squared = 88.269; Figure S10. Effect size and standardized mean difference for WHR in people supplementing fiber vs. controls. Q = 1.012, df(Q) = 1, *p* = 0.314, I-squared = 1.181; Figure S11. Funnel plot for endpoint energy intake (SMD) in present meta-analysis. Egger’s test: *p* = 0.035; Figure S12. Funnel plot for endpoint LBM (SMD) in present meta-analysis. Egger’s test: *p* = 0.094; Figure S13. Funnel plot for endpoint MBF (SMD) in present meta-analysis. Egger’s test: *p* = 0.346; Figure S14. Funnel plot for endpoint PBF (SMD) in present meta-analysis. Egger’s test: *p* = 0.100; Figure S15. Funnel plot for endpoint Body weight (SMD) in present meta-analysis. Egger’s test: *p* = 0.074.
