# Supplementary material for: The Relationship between Prebiotic Supplementation and Anthropometric and Biochemical Parameters in Patients with NAFLD—A Systematic Review and Meta-Analysis of Randomized Controlled Trials
Source: Nutrients. 2020 Nov 11;12(11):3460. doi: 10.3390/nu12113460 (PMC7698299; doi:10.3390/nu12113460)
Supplement: Supplementary file 1 [file nutrients-12-03460-s001.pdf]

Supplementary figures

Carbohydrate intake

Model	Study name	Std diff in means	Standard error	Variance	Lower limit	Upper limit	Z-Value	p-Value
	Akbarzadeh et al., 2016	-0,386	0,233	0,054	-0,842	0,071	-1,654	0,098
	Javadi et al., 2018	0,981	0,343	0,118	0,308	1,654	2,856	0,004
Fixed		0,045	0,193	0,037	-0,333	0,423	0,235	0,814
Random		0,274	0,683	0,466	-1,064	1,612	0,402	0,688

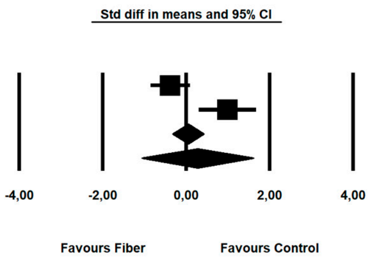

Figure S1. An effect size, standardized mean difference, for carbohydrate intake in persons supplementing fiber vs. controls. Q = 10.836, df(Q) = 1, *p* =0.001, I-squared = 90.771.

Energy intake

Model	Study name	Std diff in means	Standard error	Variance	Lower limit	Upper limit	Z-Value	p-Value
	Akbarzadeh et al., 2016	-0,238	0,232	0,054	-0,692	0,216	-1,026	0,305
	Behrouzet al.., 2017	0,018	0,260	0,068	-0,492	0,528	0,069	0,945
	Javadi et al., 2018	0,523	0,330	0,109	-0,124	1,170	1,585	0,113
Fixed		0,015	0,153	0,024	-0,285	0,316	0,098	0,922
Random		0,050	0,208	0,043	-0,358	0,458	0,239	0,811

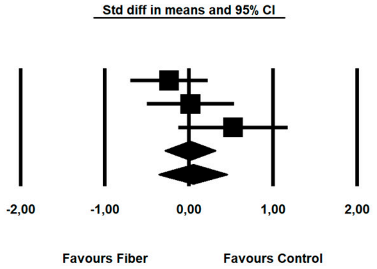

Figure S2. An effect size, standardized mean difference, for energy intake in persons supplementing fiber vs. controls. Q = 3.560, df(Q) = 2, *p* =0.169, I-squared = 43.825.

Fat intake

Model	Study name	Std diff in means	Standard error	Variance	Lower limit	Upper limit	Z-Value	p-Value
	Akbarzadeh et al., 2016	-0,160	0,231	0,054	-0,613	0,294	-0,691	0,490
	Javadi et al., 2018	-0,640	0,333	0,111	-1,292	0,012	-1,924	0,054
Fixed		-0,316	0,190	0,036	-0,689	0,056	-1,666	0,096
Random		-0,340	0,233	0,054	-0,796	0,115	-1,463	0,143

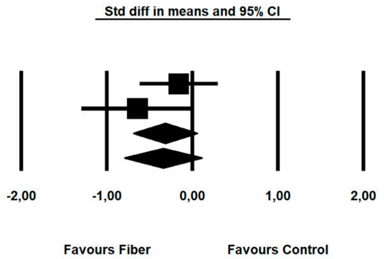

Figure S3. An effect size, standardized mean difference, for fat intake in persons supplementing fiber vs. controls. Q = 1.404, df(Q) =1, *p* =0.236, I-squared = 28.724.

LBM

Model	Study name	Std diff in means	Standard error	Variance	Lower limit	Upper limit	Z-Value	p-Value
	Akbarian et al., 2016	3,166	0,501	0,251	2,185	4,147	6,323	0,000
	Akbarzadeh et al., 2016	-0,553	0,235	0,055	-1,014	-0,092	-2,350	0,019
	Javadi et al., 2018	0,326	0,327	0,107	-0,314	0,966	0,999	0,318
Fixed		0,181	0,178	0,032	-0,168	0,531	1,017	0,309
Random		0,930	0,928	0,862	-0,890	2,749	1,002	0,317

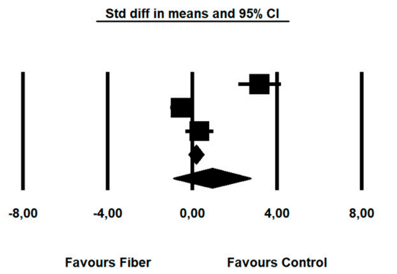

Figure S4. An effect size, standardized mean difference, for LBM in persons supplementing fiber vs. controls. Q = 45.474, df(Q) = 2, p =0.00, I-squared = 95.602.

MBF

Model	Study name	Std diff in means	Standard error	Variance	Lower limit	Upper limit	Z-Value	p-Value
	Akbarian et al., 2016	-0,699	0,344	0,118	-1,373	-0,025	-2,032	0,042
	Akbarzadeh et al., 2016	0,969	0,435	0,189	0,116	1,821	2,226	0,026
	Javadi et al., 2018	-0,202	0,325	0,106	-0,839	0,436	-0,621	0,535
Fixed		-0,117	0,208	0,043	-0,524	0,290	-0,561	0,575
Random		-0,014	0,451	0,204	-0,898	0,871	-0,030	0,976

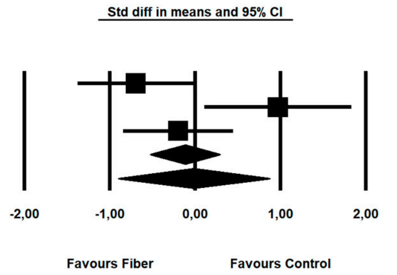

Figure S5. An effect size, standardized mean difference, for MBF in persons supplementing fiber vs. controls. Q = 9.158, df(Q) = 2, p =0.01, I-squared = 78.161.

PBF

Model	Study name	Std diff in means	Standard error	Variance	Lower limit	Upper limit	Z-Value	p-Value
	Akbarian et al., 2016	-2,358	0,434	0,189	-3,209	-1,506	-5,428	0,000
	Akbarzadeh et al., 2016	-0,182	0,231	0,054	-0,635	0,272	-0,784	0,433
	Behrouzet al., 2017	-0,264	0,262	0,068	-0,776	0,249	-1,008	0,313
Fixed		-0,512	0,161	0,026	-0,827	-0,196	-3,178	0,001
Random		-0,871	0,552	0,305	-1,954	0,212	-1,577	0,115

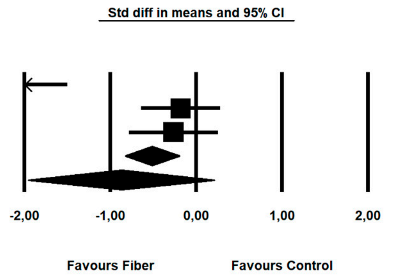

Figure S6. An effect size, standardized mean difference, for PBF in persons supplementing fiber vs. controls. Q = 20.996, df(Q) = 2, p =0.00, I-squared = 90.474.

Protein intake

Model	Study name	Std diff in means	Standard error	Variance	Lower limit	Upper limit	Z-Value	p-Value
	Akbarzadeh et al., 2016	0,151	0,231	0,053	-0,303	0,604	0,651	0,515
	Javadi et al., 2018	0,591	0,331	0,110	-0,058	1,241	1,784	0,074
Fixed		0,295	0,190	0,036	-0,077	0,667	1,555	0,120
Random		0,307	0,211	0,045	-0,106	0,721	1,455	0,146

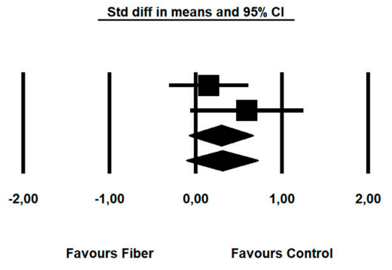

Figure S7. An effect size, standardized mean difference, for protein intake in persons supplementing fiber vs. controls. Q = 1.190, df(Q) = 1, p =0.275, I-squared = 15.955.

SLM

Model	Study name	Std diff in means	Standard error	Variance	Lower limit	Upper limit	Z-Value	p-Value
	Akbarian et al., 2016	3,278	0,511	0,261	2,277	4,279	6,420	0,000
	Akbarzadeh et al., 2016	-0,544	0,235	0,055	-1,005	-0,083	-2,313	0,021
Fixed		0,125	0,214	0,046	-0,294	0,544	0,585	0,558
Random		1,340	1,911	3,652	-2,405	5,085	0,701	0,483

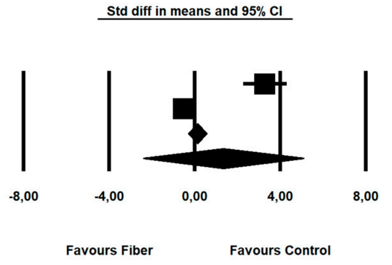

Figure S8. An effect size, standardized mean difference, for SLM in persons supplementing fiber vs. controls. Q = 46.227, df(Q) = 1, p =0.00, I-squared = 97.837.

Body weight

Model	Study name	Std diff in means	Standard error	Variance	Lower limit	Upper limit	Z-Value	p-Value
	Akbarian et al., 2016	1,397	0,372	0,139	0,668	2,127	3,754	0,000
	Akbarzadeh et al., 2016	-0,835	0,241	0,058	-1,306	-0,363	-3,466	0,001
	Behrouzet al.., 2017	-0,191	0,261	0,068	-0,702	0,321	-0,731	0,465
	Javadi et al., 2018	-0,009	0,324	0,105	-0,645	0,627	-0,027	0,978
Fixed		-0,148	0,143	0,021	-0,429	0,133	-1,031	0,302
Random		0,058	0,428	0,183	-0,781	0,897	0,136	0,892

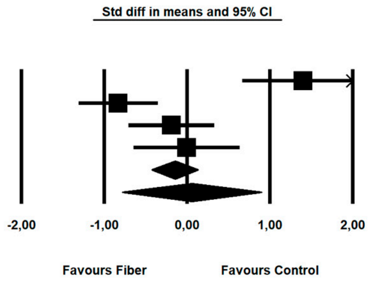

Figure S9. An effect size, standardized mean difference, for body weight in persons supplementing fiber vs. controls. Q = 25.572, df(Q) = 3, p =0.00, I-squared = 88.269.

WHR

Model	Study name	Std diff in means	Standard error	Variance	Lower limit	Upper limit	Z-Value	p-Value
	Akbarzadeh et al., 2016	-0,181	0,231	0,054	-0,634	0,273	-0,781	0,435
	Javadi et al., 2018	0,221	0,325	0,106	-0,417	0,859	0,679	0,497
Fixed		-0,046	0,189	0,036	-0,416	0,324	-0,243	0,808
Random		-0,045	0,190	0,036	-0,418	0,327	-0,238	0,812

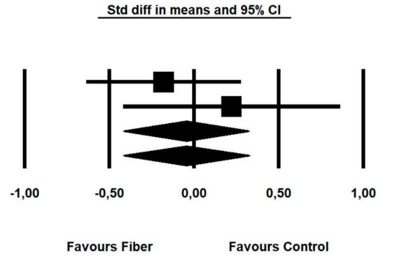

Figure S10. An effect size, standardized mean difference, for WHR in persons supplementing fiber vs. controls. Q = 1.012, df(Q) = 1, p =0.314, I-squared = 1.181.

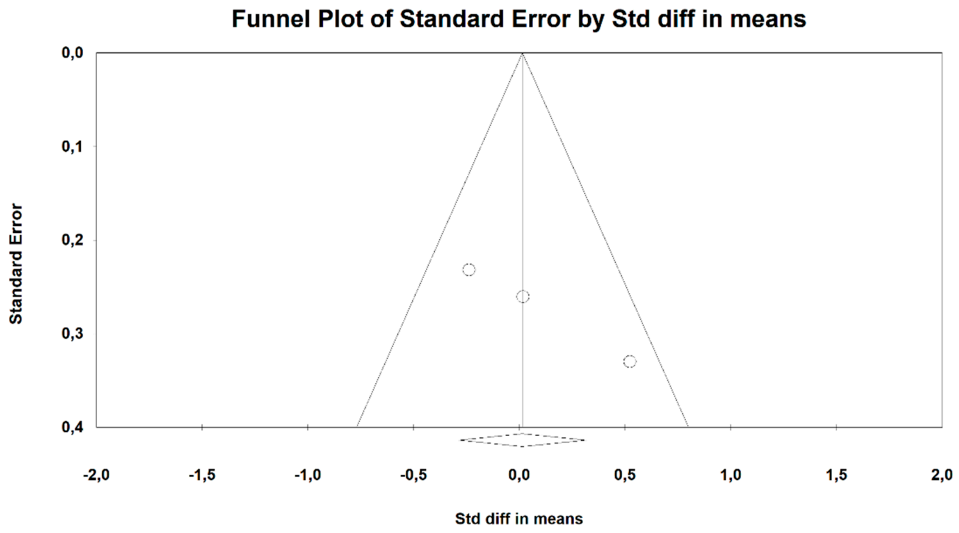

Figure S11. Funnel plot for endpoint energy intake (SMD) in present meta-analysis. Egger's test: p = 0.035

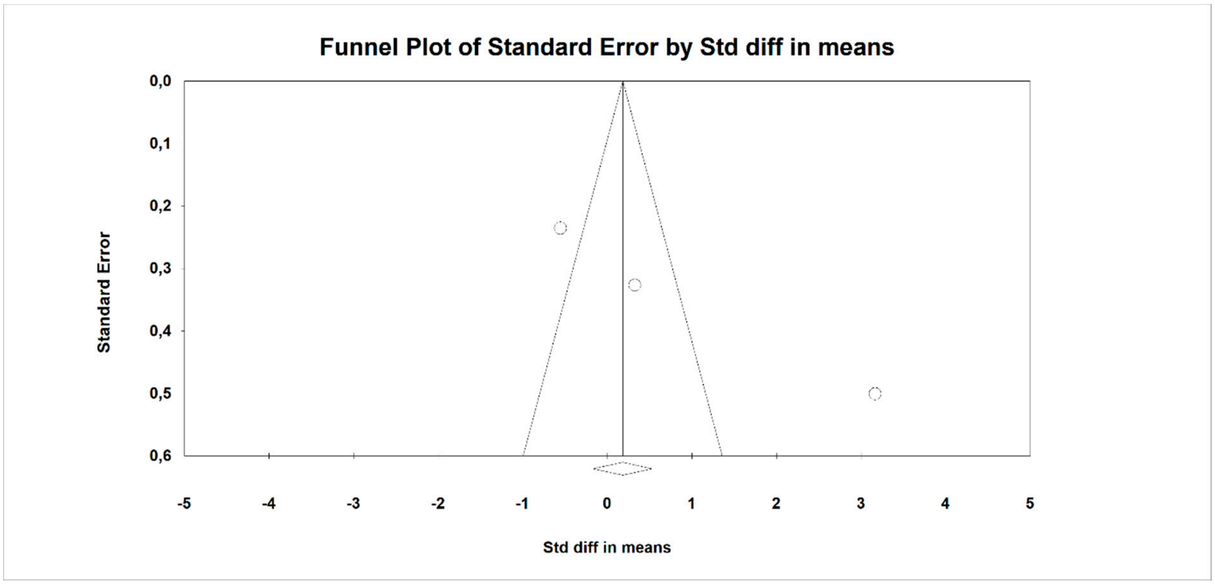

Figure S12. Funnel plot for endpoint LBM (SMD) in present meta-analysis. Egger's test: $p = 0.094$

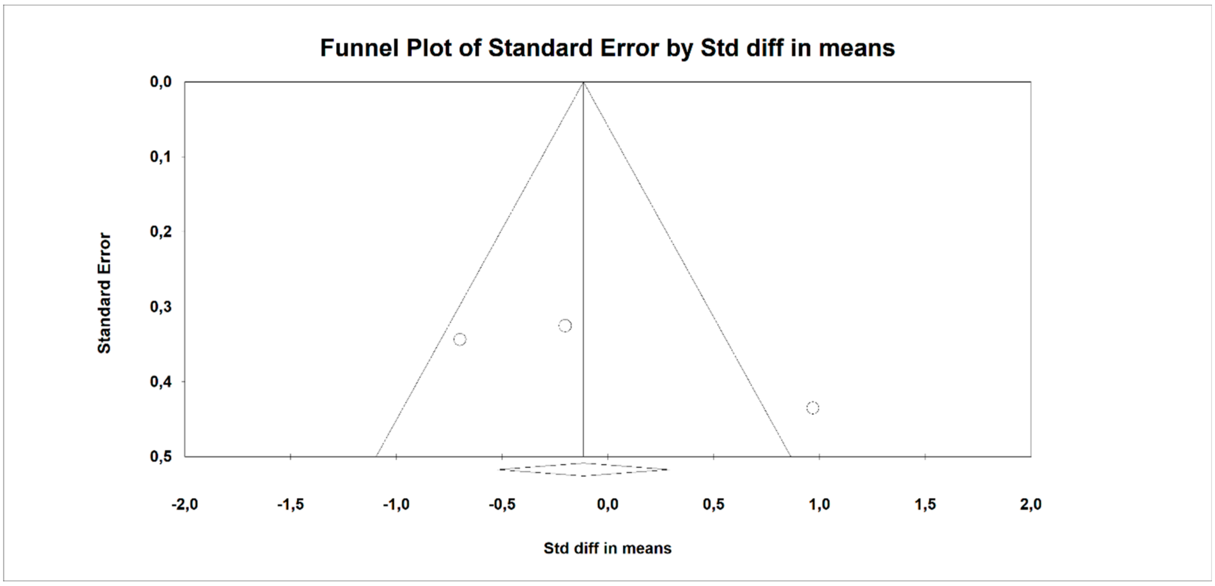

Figure S13. Funnel plot for endpoint MBF (SMD) in present meta-analysis. Egger's test: $p = 0.346$

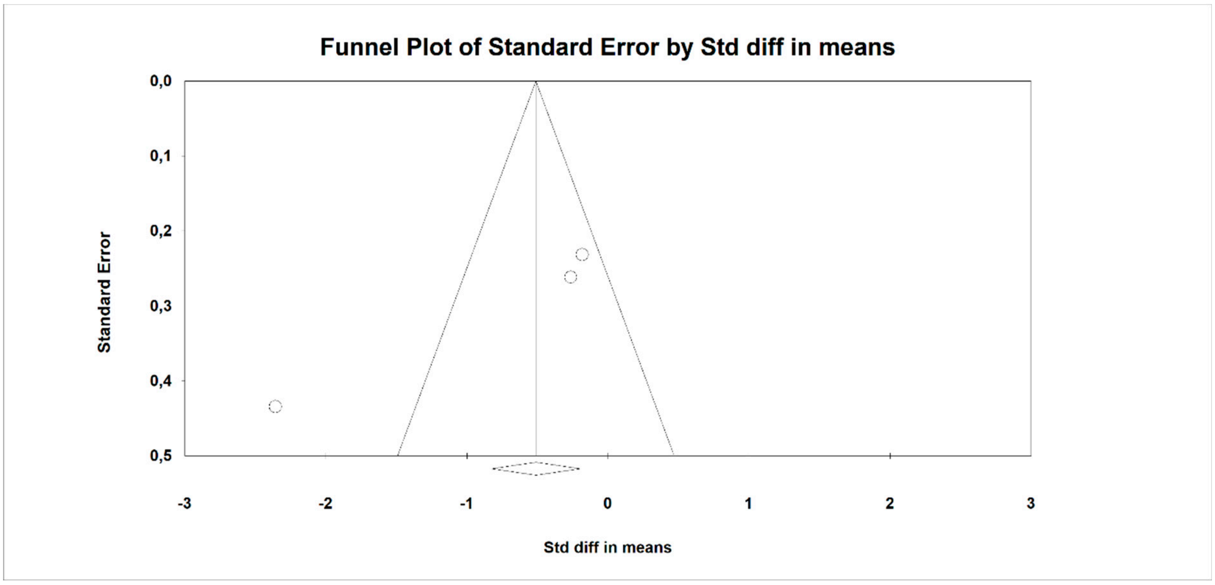

Figure S14. Funnel plot for endpoint PBF (SMD) in present meta-analysis. Egger's test: $p = 0.100$

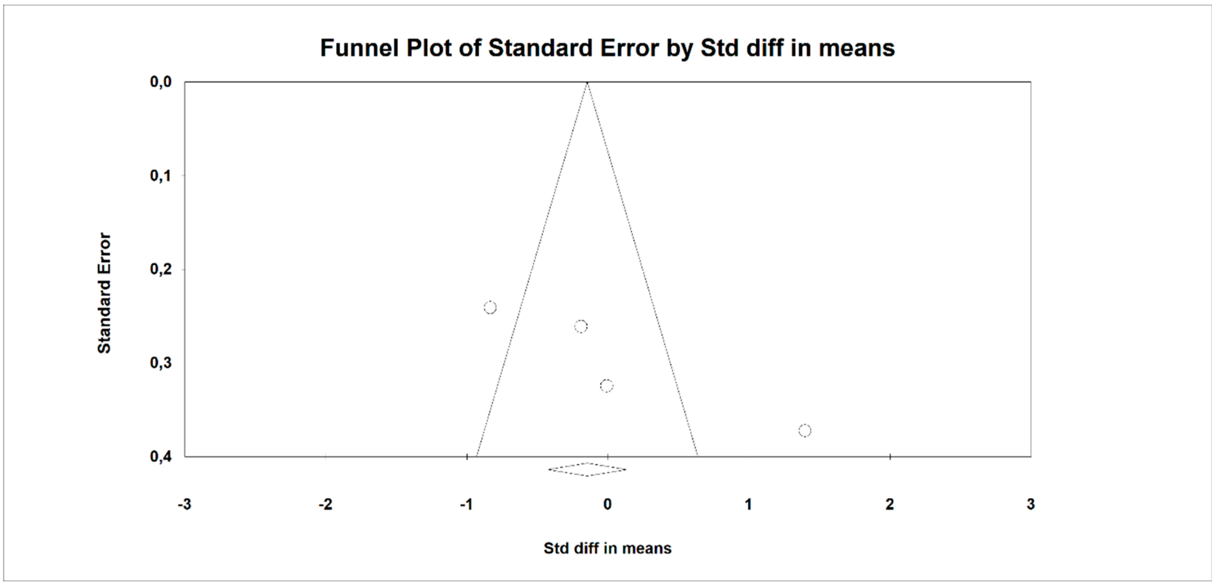

Figure S15. Funnel plot for endpoint Body weight (SMD) in present meta-analysis. Egger's test: $p = 0.074$
